# Elemental characterisation of melanin in feathers via synchrotron X-ray imaging and absorption spectroscopy

**DOI:** 10.1038/srep34002

**Published:** 2016-09-23

**Authors:** Nicholas P. Edwards, Arjen van Veelen, Jennifer Anné, Phillip L. Manning, Uwe Bergmann, William I. Sellers, Victoria M. Egerton, Dimosthenis Sokaras, Roberto Alonso-Mori, Kazumasa Wakamatsu, Shosuke Ito, Roy A. Wogelius

**Affiliations:** 1 University of Manchester, School of Earth and Environmental Sciences, Williamson Research Centre for Molecular Environmental Science, M13 9PL, UK; 2 University of Manchester, School of Earth and Environmental Sciences, Interdisciplinary Centre for Ancient Life, Manchester M13 9PL, UK; 3 College of Charleston, Department of Geology and Environmental Geosciences, Charleston, SC, 29424, USA; 4 Stanford PULSE Institute, SLAC National Accelerator Laboratory, Menlo Park, CA, 94025, USA; 5 Stanford Synchrotron Radiation Lightsource, SLAC National Accelerator Laboratory, Menlo Park, CA 94025, USA; 6 Linac Coherent Lightsource, SLAC National Accelerator Laboratory, Menlo Park, CA, 94025, USA; 7 Department of Chemistry, Fujita Health University, School of Health Sciences, Toyoake, Aichi 470–1192, Japan

## Abstract

Melanin is a critical component of biological systems, but the exact chemistry of melanin is still imprecisely known. This is partly due to melanin’s complex heterogeneous nature and partly because many studies use synthetic analogues and/or pigments extracted from their natural biological setting, which may display important differences from endogenous pigments. Here we demonstrate how synchrotron X-ray analyses can non-destructively characterise the elements associated with melanin pigment *in situ* within extant feathers. Elemental imaging shows that the distributions of Ca, Cu and Zn are almost exclusively controlled by melanin pigment distribution. X-ray absorption spectroscopy demonstrates that the atomic coordination of zinc and sulfur is different within eumelanised regions compared to pheomelanised regions. This not only impacts our fundamental understanding of pigmentation in extant organisms but also provides a significant contribution to the evidence-based colour palette available for reconstructing the appearance of fossil organisms.

Melanin pigment is one of the most widespread mechanisms in the colouration of biological tissues, most familiar as the black/brown (eumelanin) and red/yellow (pheomelanin) hues in keratinous integumentary structures such as skin, hair and feathers^1^,^2^,^3^,^4^,^5^,^6^. Melanin not only imparts colour but is also involved in a wide range of physiological processes, performing as a photo-protector^1^,^7^,^8^,^9^, contributing to elemental homeostasis^1^,^3^,^10^,^11^, and playing a role in diseases such as melanoma and Parkinson’s^11^,^12^. The role of metal ions in melanogenesis and the affinity of melanin for metal ions are of particular scientific interest. The production of melanin is catalysed by the copper coordinated enzyme tyrosinase, the genetic mutation of which causes albinism^13^. Furthermore, changes in the concentrations of copper and other metal ions have been shown to influence the melanogenesis biochemical pathway thus resulting in changes to melanin pigment production^8^,^14^,^15^. Studies have repeatedly shown that certain elements (such as Fe, Ca, Cu and Zn) are enriched within pigmented tissues compared to non-pigmented tissues^3^,^10^,^11^,^16^,^17^,^18^ which is consistent with the proposed roles of melanin as a metal reservoir (storage and release of elements) and as a metal sink (sequestration of toxic elements)^19^.

However, there are aspects of these interactions that are still unclear. Melanin is notoriously difficult to characterise due to its insolubility and heterogeneous structure^2^,^3^,^4^,^5^,^6^. Additionally, most studies have relied upon studying synthetic analogues, bulk analysis, and/or aggressive extraction methods that release melanin/melanosomes from their natural biological setting, all of which have unknown effects on the properties of the melanin molecule^18^,^20^,^21^,^22^,^23^,^24^. Studies are also difficult to compare due to the wide range of variables that can influence melanin production, characteristics, and loading in tissues. In particular, despite melanin being synthesised within the body^1^,^8^,^13^, the concentration and distribution of melanin is consistently shown to correlate with certain metal ions, the bioavailability of which are controlled by environmental exposure and dietary uptake. However, a host of other factors can affect elemental loading in tissues that might be unrelated to melanin production, such as species, sex, age, health and the aforementioned environmental/dietary factors (inc. differences between domestic, laboratory and wild animals). Finally, there is a general lack of data correlating elemental inventory with pigment variation due to the limitation of most conventional chemical analyses that are unable to spatially resolve elemental distributions that often vary over large areas.

A relatively new methodology could provide new insight into melanin and its associated metallome. Wogelius *et al*.^17^ employed Synchrotron Rapid Scanning X-ray fluorescence (SRS-XRF) imaging and X-ray absorption spectroscopy (XAS) to characterise and spatially resolve the trace metal inventory of extant and fossil tissues. A key novelty of this study was the ability to map the distribution of the elements within biological tissues on the decimetre scale at sub-millimetre resolution and to determine the coordination environment of those elements *in-situ*. Large scale imaging showed certain elements (Ca, Cu, Zn and S) to be constrained within discrete extant and fossil biological structures (feathers, fish eye and cephalopod ink sack). XAS showed that in fossil tissues Cu was bonded to organic compounds, with a coordination chemistry very similar to Cu contained within eumelanised extant analogues, eumelanin standards and in theoretical computational models for Cu in eumelanin^17^,^25^. Therefore it was interpreted that the distribution of organically complexed trace metals (Cu especially) could act as a biomarker for eumelanin distribution in extinct specimens from the fossil record. From this the first pigment reconstruction of an extinct organism (a 120 million year old bird) based on whole organism analysis was presented^17^.

However, this work focused on eumelanin because there was insufficient data at the time to confidently identify and differentiate the equally significant pheomelanin. Here we attempt to further characterise both eumelanin and pheomelanin using synchrotron X-ray techniques so that we may better constrain the nature of melanin *in situ* within tissues, and also provide further information that will allow more accurate pigment reconstruction within fossils. In the current study we analysed extant feathers from four genera of birds of prey - Harris hawk (*Parabuteo unicinctus*), red-tailed hawk (*Buteo jamaicensis*), kestrel (*Falco sparverius, Falco tinnunculus*) and barn owl (*Tyto alba*) (Supplementary Fig. 1). These species were chosen as they demonstrate a wide range of colouration from bright reds to browns and blacks, but also include unpigmented regions and strong pigment patterns within single feathers. These species are also globally widespread and therefore readily available for sampling. Furthermore, we focused on individual, strongly patterned, and mixed melanin feathers so that we could minimize the effects of a number of variables that could influence elemental loading in such tissues, such as naturally occurring variations between individual feathers, individual animals or species.

## Results

### Melanin identification

**Figure 1:**
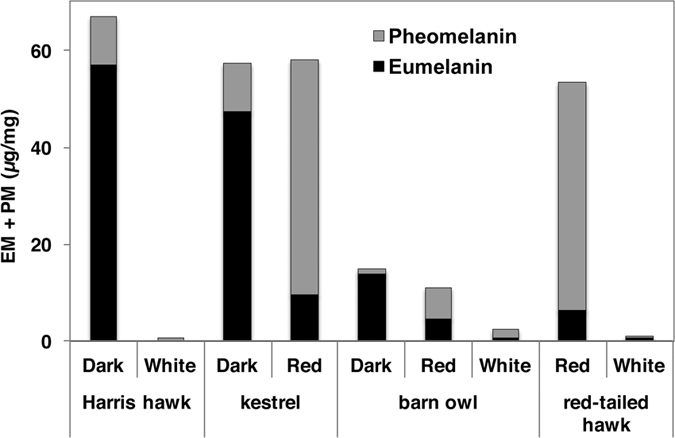
Bar graph of melanin identification and quantification from Harris hawk, Eurasian kestrel, barn owl and red-tailed hawk (UK specimens).

Melanin identification and quantification was performed according to published procedures^26^ (Fig. 1 and Supplementary Table 1). Results from the feathers were consistent with previously published results for human hair^26^ showing that pigmented regions are in fact a mixture of both eu- and pheomelanin. Results also confirmed that visibly black regions of the feathers are predominantly eumelanin pigmented, that red regions are predominantly pheomelanin pigmented, and that white regions contained very little or undetectable levels of melanin, but if pigment were detected, white regions contained more pheomelanin than eumelanin. As such, feather colour will be from hereon referred to as eumelanised and pheomelanised for black/dark and red respectively. Also of note is that total melanin content is widely variable.

### SRS-XRF

**Figure 2:**
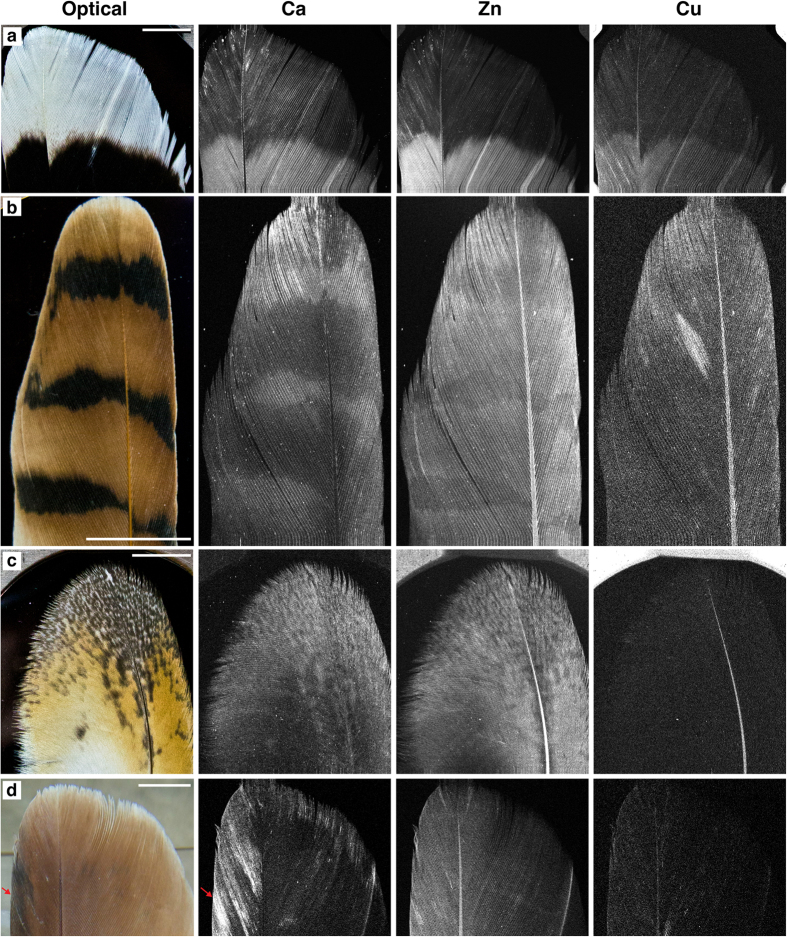
Optical images and SRS-XRF of Ca, Zn and Cu distributions in extant feathers of (**a**) Harris hawk, (**b**) kestrel (**c**) barn owl and (**d**) red-tailed hawk. These images clearly show that these elements are controlled by pigment patterns. Note that higher brightness represents higher concentrations (photon counts) and that each element map is independently scaled (8-bit, 98th percentile). Pixel size = 50 microns. Map acquisition time = ~30–40 mins. Scale bars = 1 cm.

SRS-XRF elemental maps of Harris hawk, kestrel, barn owl and red-tailed hawk feathers were obtained (Fig. 2 and Supplementary Fig. 2). These images unequivocally show that the distribution of Ca, Zn and Cu in these feathers correlate with visible pigment patterns. In the Harris hawk feather all three of these elements are elevated within the eumelanised region and their concentrations drop significantly across the boundary with the white region (Fig. 2a and Table 1). Patterning is also clearly resolved in the kestrel (Fig. 2b) and barn owl (Fig. 2c). However, while Ca is elevated within the eumelanised stripes and speckles of the kestrel and barn owl (respectively), Zn is depleted in eumelanised areas compared to adjacent pheomelanised areas. In the red-tailed hawk (Fig. 2d) Zn content correlates with the intensity of red pigment (intensity decreasing slightly from leading edge to trailing edge), while Ca correlates with darker areas in the leading edge (arrowhead). Additionally, Fe is elevated in the leading edges of both the Harris hawk and red-tailed hawk (Supplementary Fig. 2-1a,c respectively). Finally, in an example of striped feather from a red-tailed Hawk (Supplementary Fig. 2-1b) Ca, Zn and Cu also resolve the striped pattern. Synchrotron XRF Energy Dispersive Spectroscopy (EDS, Supplementary Figs 3 and 4) and point quantification (Table 1 and Supplementary Table 2) clearly shows the drop in elemental concentrations in unpigmented compared to melanised regions but also show that absolute values are highly variable between feathers. These concentrations are consistent with those detected via Particle-Induced X-ray Emission (PIXE) analysis^16^.

### XAS

Zn K-edge Extended X-ray Absorption Fine Structure (EXAFS) spectroscopy (Supplementary Fig. 5 and Table 2) indicates that there is a measurable distinction between eumelanised and pheomelanised pigmented tissue. Zn is predominantly present as an organic complex with 4-fold first shell coordination to O/N (CN = 4, R = ~2.0 Å) in the eumelanised Harris hawk feather. However, pheomelanised tissue in the kestrel feather clearly has S (CN = 1 at R = ~2.3 Å) either substituting for, or additional to, one of the first shell light elements (Supplementary Figs 5 and 6). First shell coordination of S with Zn is also resolved in the red portions of the barn owl feather and even in the nominally “white” area of the Harris hawk feather which has trace pheomelanin present (see Supplementary Table 1). Additionally, there is a noticeable difference in the shape and position of the peak between k 3–5 Å^−1^ of feathers with a strong eumelanin component and those with predominantly pheomelanin component (Supplementary Fig. 5). These are important and diagnostic differences in Zn coordinatindicated by arrows) the striped patternion chemistry between eumelanin and pheomelanin.

**Figure 3:**
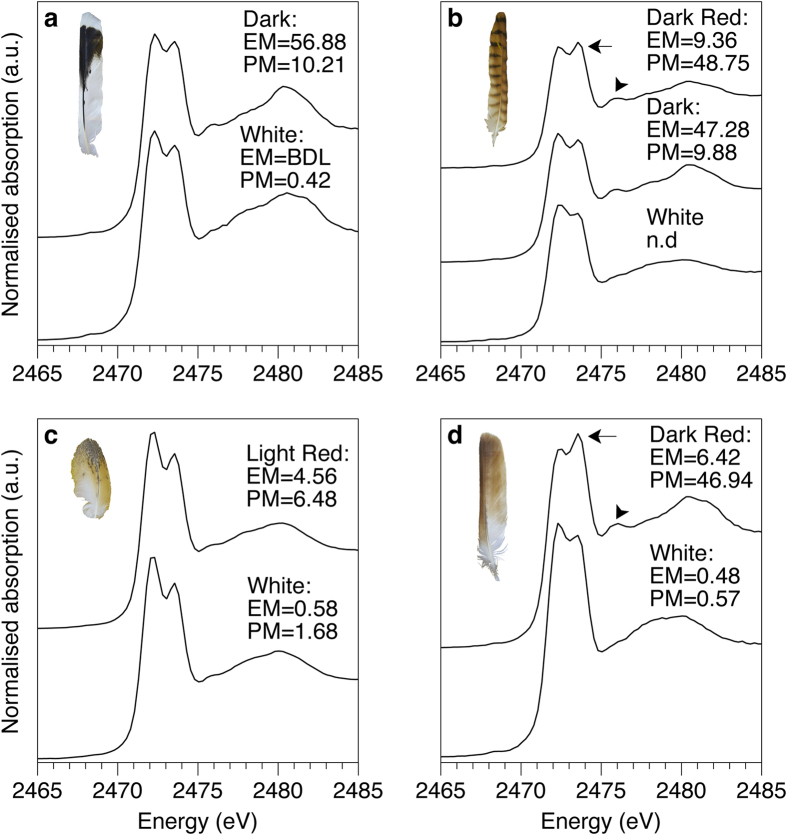
Sulfur XANES of (**a**) Harris hawk, (**b**) kestrel, (**c**) barn owl, and (**d**) red-tailed hawk. All feather spectra are dominated by the characteristic disulfide double peak originating from keratin (compared to oxidised glutathione standard, Supplementary Fig. 7). The shift in peak intensities in pheomelanised tissues are indicated by arrows (2473.5 eV) and arrowheads (2476 eV). The identity of this shifted peak is best explained by the presence of a benzothiazole type (5-membered benzo-sulfur) units within the pheomelanin structure (Supplementary Fig. 7). EM = eumelanin μg/mg, PM = pheomelanin μg/mg, BDL = below detection limits, n.d. = no data.

Sulfur X-ray Absorption Near Edge Structure (XANES) spectroscopy was also performed (Fig. 3 and Supplementary Fig. 7) in order to investigate the potential difference in S coordination between eu- and pheomelanised feathers i.e., to detect the inclusion of S in the pheomelanin molecule (as benzothiazine/zole units). The spectra of all the feathers, both melanised and unpigmented, are dominated by two sharp peaks at 2472.3 and 2473.5 eV as well as a broader feature at ~2480.4 eV. The double peaks originate from the abundant disulfide and sulfur-carbon bonds (Supplementary Fig. 7, oxidised glutathione standard) of keratin protein^27^,^28^,^29^,^30^. The key result here is a subtle difference in the spectrum of strongly pheomelanised regions of the kestrel and red-tailed hawk compared to the eumelanised and unpigmented feathers. In strongly pheomelanised feathers the distinctive double peak intensity profile of keratin seen in black and white feathers (with the 2472.3 eV peak greater than the 2473.5 eV peak) is reversed (arrows Fig. 3b,d). This almost certainly is due to the presence of benzosulfur type moieties (Supplementary Fig. 7) known to be present in the pheomelanin molecular structure^26^. In addition, the small peak at 2476 eV is also augmented in the regions with a significant pheomelanin component, again most likely due to the presence of the benzosulfur unit in pheomelanin. These spectral changes are not as strong in the pheomelanised region of the barn owl due to this feather being less melanised compared to the other feathers (Fig. 1). We conclude that there is a clear difference in both S and Zn speciation between eu- and pheomelanised tissues. Because it is resolved even when pheomelanin is only present in trace amounts, the shoulder on the Zn Fourier transformed EXAFS data at 1.85 Å is perhaps the most sensitive indicator of pheomelanin via non-destructive X-ray methods.

### XAS-SRS-XRF

**Figure 4:**
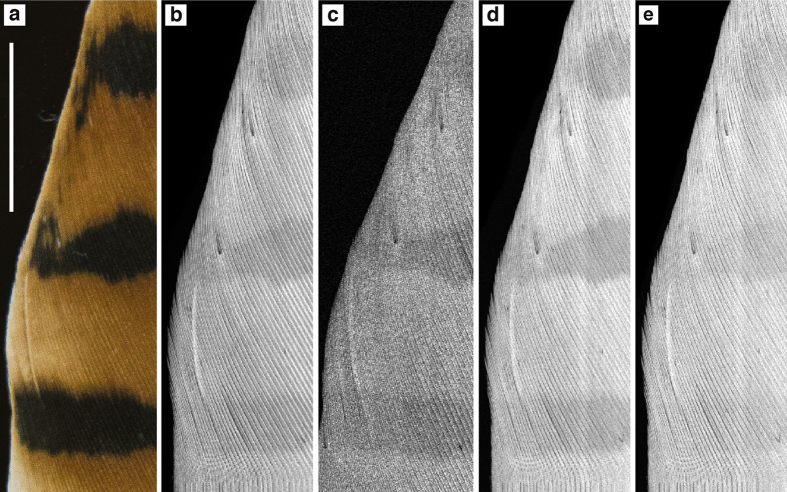
Sulfur oxidation state SRS-XRF mapping of kestrel feather. (**a**) Optical, (**b**) total sulfur (3150 eV), (**c**) 2476 eV, (**d**) 2473.5 eV, (**e**) 2472.3 eV. The total sulfur image shows that sulfur is elevated within and appears to correlate with red pigment compared to the dark stripes. Additionally, the distribution of specific oxidation states are not directly correlated. In particular, the striped pattern is more distinct in c and d compared to e. Pixel size = 50 microns. Map acquisition time = ~20 mins. Scale bar = 1 cm.

SRS-XRF imaging is able to map the distribution of specific oxidation states of an element within a sample by tuning the energy of the incident X-ray beam to specific XANES resonances. This ability allows S speciation and distribution to be better resolved, as data are not restricted to localised spectra from point analyses^31^. A map of the kestrel feather (Fig. 4a) was obtained with an incident X-ray beam energy of 3150 eV (Fig. 4b) inducing X-ray fluorescence of all oxidation states of S. This map reveals that despite the high sulfur content of keratin, the contribution of S from pigment is strong enough to be resolved, in particular that S is elevated in the pheomelanised regions compared to the eumelanised stripes. Maps obtained using incident energies matching that of the individual S peaks are presented in Fig. 4c (2476 eV), 4d (2473.5 eV) and 4 e (2472.3 eV). In the maps of the benzosulfur resonances (Fig. 4c,d; also see Fig. 3b,d indicated by arrows) the striped pattern is more distinct than that seen in the total S map (Fig. 4b) as well as more distinct than the map obtained at 2472.3 eV (disulfide, Fig. 4e). We conclude that pheomelanin associated S can be mapped in extant tissue with this method.

## Discussion

SRS-XRF unequivocally shows that the distribution of Ca, Cu, and Zn, is controlled by melanin pigment, which is consistent with other studies^16^,^17^,^18^,^23^. Ca is common to eumelanised regions in all feathers analysed, and as such could act as a potential marker for eumelanin. Zn is also clearly enriched within eumelanised regions of the Harris hawk and striped red-tailed hawk (Fig. 2a and Supplementary Fig. 2-1b). However, in the eumelanised stripes and speckles of the kestrel and barn owl (Fig. 2b and c respectively) Zn appears depleted compared to the adjacent pheomelanised regions. In the kestrel, total melanin concentration is nearly identical in the eumelanised and pheomelanised regions. This indicates that Zn has a higher affinity for pheomelanin. Cu also appears to be uniquely present within eumelanin, but the contrast between eumelanised and unpigmented regions is significantly weaker for some species than that seen for Ca and Zn, being indiscernible in the kestrel and barn owl (Fig. 2b,c). Imaging indicates that the combined presence of Ca, Zn and Cu compared to unpigmented regions is indicative of a predominantly eumelanin content and that Zn in isolation is indicative of a predominantly pheomelanin content. Eumelanin/pheomelanin except for the white feather was shown to co-exist as indicated in Supplementary Table 1. Recently Gorniak *et al*.^22^ indicated that Cu specifically associated with eumelanin is more likely to be found close to the surface of melanosomes, and Ca and Zn are concentrated within their core regions. This supports the casing model that predicts individual melanosomes to contain a pheomelanin core surrounded by a eumelanin shell. Thus, the analysis of variable concentrations and distributions of metal ions, Cu, Ca and Zn, may contribute to understanding which types of melanin may co-exist. On the other hand, the increases in intracellular Ca^2+^ elicit changes in cellular melanin contents^32^, and the Na^+^/Ca^2+^ exchanger activity of SLC24A5^33^ may provide a link between cytosolic and melanosomal Ca^2+^ signaling by regulating Ca^2+^ transport from cytosol to melanosome lumen.

Elemental quantification corroborates the imaging data but shows that quantification can be ambiguous if used in isolation. For example, there are similar levels of Zn in the unpigmented Harris hawk feather (37 ppm) and the pheomelanised red-tailed hawk (32 ppm) and kestrel feather (49 ppm). Additionally, the pheomelanised regions of the barn owl shows higher levels of Zn than its eumelanised speckles, but these differences are only on the order of a few ppm (4 ppm). It is these minute differences in concentration that demonstrate the importance of imaging in being able to resolve element patterns. If point analysis quantification was taken in isolation, one might conclude there is no distinct correlation between pigment patterns and elemental inventory in many of the cases presented above. Figures 2 and 4 show that imaging is critical in understanding pigment affiliated element distributions because it is relative concentration, not absolute concentrations, that correlates with pigment patterns. The results from this study do not indicate any species specific metal/melanin associations. However, we did not analyse a statistically significant number of feathers from each species to be able to test changes in metal/melanin content with species. Highly constrained studies on species specific variations have not previously been conducted but variations that have been observed are often ambiguous due to the other variables that can influence metal loading in feathers from the natural habitat of different species such as genetic background, environmental exposure and diet.

XAS clearly resolved the different bonding configuration for Zn between eumelanised and pheomelanised feathers. The key difference for pheomelanin is the Zn-S bonding in the first shell, compared to only Zn-N/O in eumelanin. Studies on eumelanin have found metals to be predominantly bound to oxygen, either to oxygens within the abundant carboxyl groups or to hydroxyl groups. Experimental diffraction work on trace metals in pheomelanin has however suggested that the coordination of trace metals to S can occur in pheomelanogenesis^34^,^35^,^36^. A comparison of our Zn-EXAFS results for the eumelanin standard with the pheomelanised feathers agrees with these previous results, and shows conclusively via *in situ* analysis that the Zn coordination chemistry in pheomelanin is different from that in eumelanin. Interestingly, XAS results of the white region of the Harris hawk feather shows a very similar bonding configuration to pheomelanised feathers (Table 2 and Supplementary Fig. 5, a slight broadening of the central peak) which is consistent with the minute quantities of pheomelanin (but undetectable eumelanin) measured in this region (Fig. 1).

We also note that the data here are wholly inconsistent with Zn coordinated to keratin in either eu- or pheomelanised feathers^37^, because if a significant portion of the metals were bound to keratin, this bonding mode would surely dominate the EXAFS signal and no difference would be seen between the eu- and pheomelanised regions. Furthermore, the metal distributions seen in Fig. 2 would have to be explained by another mechanism of metal deposition in integumentary structures, but such a mechanism would be unlikely to correlate with pigment patterns.

XAS also resolves a significant but subtly different bonding environment for S between pheomelanised and unpigmented tissue. The intensity shift in the double peak at 2473.5 eV and the peak at 2476 eV is most likely due to the presence of benzosulfur units in the pheomelanin structure (Supplementary Fig. 7). The double peak intensity shift only seems to occur in heavily pheomelanised feathers. If pheomelanin concentration is relatively low overall (as in the barn owl) the contribution of benzosulfur units to the peak at 2473.5 eV is overwhelmed by the signal from keratin. However, the peak at 2476 eV is not affected by the dominating keratin signal and thus is a more sensitive indicator for the presence of pheomelanin in a biological matrix than the peak at 2473.5 eV. The presence of benzosulfur within pheomelanised regions would be expected because benzothiazole is one of the important end products of the pheomelanogenesis pathway and part of the structure of pheomelanin^34^,^35^. We propose that the S exposed in the benzothiazole ring can form inner sphere complexes with metals such as Zn in pheomelanin-rich tissues, thus explaining the S and Zn spectroscopy results as well as the large-scale trace element and oxidation state maps.

This study shows that synchrotron X-ray analyses can be used to non-destructively identify, map, and characterise the elemental inventory and chemical coordination environments of melanised tissues. The ability to rapidly map on the decimetre scale at sub-millimetre resolution greatly improves our understanding of the controls on element distributions in biological tissues. Here, our comparison of large scale elemental distributions with optically visible colouration unequivocally show that the presence and distribution of Ca, Cu and Zn are controlled by melanin pigment patterns in feathers, whereas other elements show no obvious correlation with melanin pigment. This finding sets a benchmark for the analysis of elements associated with melanin. This study also shows how the use of chemical mapping significantly improves our understanding of the pigment patterns in biological tissues over non-spatial bulk elemental quantification. Comparisons based on single point quantification can be unreliable due to: 1) potential influences from contaminants, 2) metal/melanin concentration variability within individual feathers, and 3) different pigment levels within feathers from different species. XAS of both Zn and S revealed that melanin type can be differentiated *in situ,* an advantage over those analytical techniques that require extraction/breakdown of melanin from its natural environment, and that pheomelanin potentially possesses a distinct and detectable XAS fingerprint, a detail that was unexpected at the onset of this study. It is also apparent that the presence of elevated S concentrations in conjunction with the presence of organic Zn complexes act together as a marker for pheomelanin. Finally, elemental inventories and S oxidation states within melanised tissue have not previously been mapped at the decimetre scale: our results conclusively show that there is a resolvable chemical zoning in S and Zn that correlates with pheomelanin. These results have important implications for the analysis of pigment in a wide range of disciplines, from tissue analysis related to health issues, through to the analysis of ancient palaeontological specimens. Synchrotron X-ray analyses may now be used to produce more accurate and reliable pigment reconstructions in fossil organisms, including cases where the optical and structural fidelity of the biological tissues has been compromised through degradation processes.

## Methods

### Samples

Feathers analysed at SSRL - Harris hawk (*Parabuteo unicinctus*), red-tailed hawk (*Buteo jamaicensis*), American kestrel (*Falco sparverius*) and barn owl (*Tyto alba*) (Fig. 2 and Supplementary Fig. 1) - were obtained from animals under the care of the Live Animal Center (LAC) at The Academy of Natural Sciences of Drexel University (Philadelphia, PA). The feathers were naturally dropped via moulting and collected by staff of the LAC from the enclosures. Feathers analysed at DLS and Fujita Health University, School of Health Sciences, Japan - Harris hawk (*Parabuteo unicinctus*), Eurasian kestrel (*Falco tinnunculus*) and barn owl (*Tyto alba*) were obtained from animals under the care of the non-profit Wild Wings Birds of Prey education and rehabilitation centre located in Bents Home and Garden, Warrington, UK. Red-tailed hawk (*Buteo jamaicensis*) feathers were obtained from The Falconry Centre, Hagley, UK. The feathers were naturally dropped via moulting and collected by staff from the enclosures. In accordance with Federal regulations, all appropriate United States Department of Agriculture – Animal and Plant Health Inspection Service (USDA-APHIS) permits for keeping birds of prey and transporting feathers across state lines (permit no. 44877) to other research institutions are held by the Live Animal Center (LAC) and the ornithology department at The Academy of Natural Sciences of Drexel University (Philadelphia, PA, USA). Appropriate CITES permits from the Animal Health and Veterinary Laboratory Agency (AHVLA) were also acquired for export/import of UK sourced feathers to Japan. Wild Wings Birds of Prey education and rehabilitation centre (UK) also holds appropriate permits for keeping these species.

All feathers in this study were stored in sealed bags and stored in a freezer until analysis but were subjected to no other treatment or preparation other than being blown with compressed air to remove any surface particulates. Note that the results in Fig. 1 and Supplementary Fig. 5 were sourced from Eurasian kestrel as opposed to an American kestrel in Fig. 2. Due to the expense of and time constraints on synchrotron beam access, the number of samples that can be analysed is severely limited. Thus in order to meet the objectives of the experiment within those time constraints, we prioritised analyzing feathers from different species over analyzing multiple feathers from a single animal or species. The elemental mapping, quantification and XAS data from SSRL were all obtained from one feather for each of the four species from the LAC (USA). The Zn EXAFS data from DLS were again obtained from one feather of each of the four species from the Wild Wings Birds of Prey education and rehabilitation centre (UK). The melanin quantifications presented in Fig. 1 were from a further four feathers of each of the specified species from the Wild Wings Birds of Prey education and rehabilitation centre (UK). The Zn doped eumelanin standard was produced by washing 50 mg of *Sepia officinalis* melanin (Sigma Aldrich) in 50 ml of 0.1 mol^−1^ Zn sulfate (ZnSO_4_.7H_2_0) aqueous solution with a pH of approximately 6.5. This was left to stand for 24 hours then centrifuged and the supernatant removed. The precipitate was washed with deionised water which was then freeze dried for analysis^38^,^39^,^40^.

### Melanin identification and content

Melanin identification and quantification was performed as outlined in^26^. Individual feathers from the UK were sent to Fujita Health University, School of Health Sciences, Japan under the appropriate CITES documentation and licenses from the primary institute conducting this study (University of Manchester). Feathers from the USA had to remain within the USA. 2–18 mg of feather samples were dissected, and homogenized with Ten-Broeck homogenizer at a concentration of 10 mg/mL H_2_O and 100 μL (1 mg) aliquots were subjected to Soluene-350 solubilization^1^, hydroiodic acid hydrolysis^2^, and alkaline hydrogen peroxide oxidation^3^. Analyses were performed in duplicate.

### Synchrotron Imaging

Synchrotron X-ray fluorescence imaging was performed at the Stanford Synchrotron Radiation Lightsource (SSRL) wiggler beam line 6–2 at the Stanford Linear Accelerator Center (SLAC, CA, USA). Extensive and detailed descriptions of SRS-XRF mapping applied to fossils are provided in recent previous publications^41^,^42^ and so are only summarised here. Experiments were operated with an incident beam energy of either 13.5 keV (flux calculated between 10^10^ and 10^11^ photons s^−1^) or 3.15 keV (flux ~10^9^ photons s^−1^) and a beam diameter of 50 microns defined by a pinhole. X-rays were detected using a single element Vortex silicon drift detector.

### Quantification

Energy dispersive spectra obtained from SSRL were collected for 50 live seconds and fit using PyMCA^43^ from fundamental parameters of the experiment using a Durango apatite (fluoroapatite) mineral standard with known element concentrations for calibration. 2σ error calculated using the sigma area value for each element output by PyMCA (e.g. Supplementary Fig. 4). Please consult^41^,^42^ for further details. The concentrations given for each colour of each feather is an average of 3 individual 50 second EDS spectra taken within a few hundred microns of each other on the same specimen.

### X-ray Absorption Spectroscopy (XAS)

Sulfur X-ray Absorption Near Edge Structure (XANES) data were recorded at SSRL beam line 6–2 in fluorescence mode using a single element Vortex silicon drift detector set at ~60° scattering angle. S XANES were also collected at Diamond Light Source (DLS) microfocus beamline I18 in fluorescence mode using a four element Si drift Vortex fluorescence detector set at 90° scattering angle. Zn Extended X-ray Absorption Fine Structure (EXAFS) were collected at DLS beamline I18 in fluorescence mode using a four element Si drift Vortex fluorescence detector set at 90° scattering angle. ZnSO_4_ standards were used to calibrate the energy of the monochromator position, defined at 2482 eV. Data averaging, background subtraction, data normalisation and fitting of the XANES and EXAFS spectra were performed using Demeter software package^44^. In the EXAFS fits, the Hamilton and F-tests were used to verify the significance of all shells used in the fitted models^45^. Depending on the quality, a minimum of 16 spectra were acquired for each specimen to improve the signal-to-noise ratio. All S compounds analysed were at least 98% pure, purchased from Alfa Aesar or Sigma Aldrich. For health and safety, some of the sulfur compounds studied here were not pure isolated examples of S moieties of interest (e.g. 2-amino-6-chlorobenzothiazole) due to the fact that many of the isolated moieties are extremely volatile and toxic. For XAS analyses, S compounds were mixed with boron nitride to obtain a S concentration of ~5 wt% (concentrations typically used in XAS fluorescence analyses to minimise self-absorption effects).

### Image Processing and Analysis

SRS-XRF maps from SSRL were processed from the raw detector count raster files using a custom MATLAB computer script that converted the data array into viewable 8 bit tiff images clipped at various contrast percentiles.

## Additional Information

**How to cite this article**: Edwards, N. P. *et al*. Elemental characterisation of melanin in feathers via synchrotron X-ray imaging and absorption spectroscopy. *Sci. Rep.* **6**, 34002; doi: 10.1038/srep34002 (2016).

## Supplementary Material

Supplementary Information

## Figures and Tables

**Table 1:** Synchrotron XRF Energy Dispersive Spectroscopic point quantification of feathers in Fig. 2.

Species	Color	Ca	Zn	Cu	Fe	S (wt%)					
Harris hawk	Black	6421 (389)	186 (10)	12 (1)	26 (2)	3.36 (0.16)					
	White	1960 (159)	37 (3)	5 (1)	10 (1)	2.59 (0.13)					
kestrel	Black	1574 (123)	25 (2)	2 (1)	5 (1)	1.94 (0.09)					
	Red	989 (85)	49 (3)	3 (1)	5 (1)	1.36 (0.07)					
barn owl	Black	733 (73)	43 (3)	1 (1)	3 (1)	1.18 (0.05)					
	Red	423 (47)	47 (3)	1 (1)	4 (1)	2.78 (0.1)					
	White	137 (18)	25 (2)	1 (1)	3 (1)	1.21 (0.05)					
red-tailed hawk	Red	2060 (160)	32 (3)	2 (1)	8 (1)	2.89 (0.12)					
	White	35 (7)	6 (1)	0 (1)	1 (1)	2.33 (0.1)					

Values are in parts per million (ppm) apart from sulfur which is presented in weight %, number in parentheses are the 2σ errors.

**Table 2:** Zn shell by shell EXAFS fit data for UK feathers obtained from DLS I18.

sample	path	CN	R(Å)	σ^2^	ΔE_0_ (eV)	S0^2^	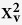	R
Harris hawk eumelanised*	Zn-O	4.1	2.01 (6)	0.009 (1)	4.142 ± 0.25	0.82 (2)	24.85	0.012
	Zn-C	0.9	2.71 (5)	0.017 (14)				
	Zn-Ca	0.9	3.42 (3)	0.011 (5)				
	Zn-O	2.1	3.84 (25)	0.009 (1)				
Eurasian kestrel pheomelanised*	Zn-O	3.0	2.02 (3)	0.012 (1)	0.283 ± 0.15	0.99 (2)	87	0.007
	**Zn-S**	0.8	2.29 (4)	0.008 (4)				
	Zn-C	2.0	2.78 (13)	0.019 (7)				
	Zn-Zn	1.2	3.34 (9)	0.021 (10)				
	Zn-O	2.8	3.75 (11)	0.017 (5)				
barn owl pheomelanised**	Zn-O	3.3	2.01 (2)	0.017 (2)	4.59 ± 1.248	1.07 (17)	5.37	0.008
	**Zn-S**	1.0	2.30 (1)	0.009 (4)				
	Zn-Ca	1.0	3.48 (2)	0.008 (3)				
Harris hawk unmelanised**	Zn-O	3.1	1.96 (2)	0.012 (2)	−0.392 ± 1.621	0.98	16.58	0.013
	Zn-S	1.5	2.26 (2)	0.009 (3)				
	Zn-Ca	1.2	3.35 (4)	0.011 (5)				
Zn eumelanin standard	Zn-O	3.9	1.98 (3)	0.012 (4)	−0.142 ± 2.066	1.02 (27)	47.340	0.022
	Zn-C	1.7	2.72 (5)	0.016 (10)				
	Zn-Zn	1.5	3.26 (6)	0.014 (6)				

Highlighted in underline bold is the distinct presence of sulfur within pheomelanised feathers. CN denotes coordination number; R(Å) denotes atomic distance; σ^2^ denotes Debye-Waller factor; ΔE_0_ denotes the shift in energy from the calculated Fermi level. S0^2^ denotes the amplitude reduction factor; *X*_*v*_^2^ denotes the reduced chi square value; R denotes the “good-ness of fit” factor. Numbers in parentheses are the 1σ error on the last decimal places. Errors on CN = 25%.

^*^Data obtained to k = 14.

^**^Low concentrations of zinc resulted in low signal to noise ratio.
