# Supplementary material for: Elemental characterisation of melanin in feathers via synchrotron X-ray imaging and absorption spectroscopy
Source: Sci Rep. 2016 Sep 23;6:34002. doi: 10.1038/srep34002 (PMC5034265; doi:10.1038/srep34002)
Supplement: Supplementary Information [file srep34002-s1.pdf]

Elemental characterisation of melanin in feathers via synchrotron X-ray imaging and absorption spectroscopy – Supplementary Information

Nicholas P. Edwards*[1,2], Arjen van Veelen[1,2], Jennifer Anné[1,2], Phillip L. Manning[2,3], Uwe Bergmann[4], William I. Sellers[1,2], Victoria M. Egerton[2,3], Dimosthenis Sokaras[5], Roberto Alonso-Mori[6], Kazumasa Wakamatsu[7], Shosuke Ito[7], Roy A. Wogelius[1,2]

[1]University of Manchester, School of Earth and Environmental Sciences, Williamson Research Centre for Molecular Environmental Science, M13 9PL, UK
[2]University of Manchester, School of Earth and Environmental Sciences, Interdisciplinary Centre for Ancient Life, Manchester M13 9PL, UK
[3]College of Charleston, Department of Geology and Environmental Geosciences, Charleston, SC, 29424, USA
[4]Stanford PULSE Institute, SLAC National Accelerator Laboratory, Menlo Park, CA, 94025, USA
[5]Stanford Synchrotron Radiation Lightsource, SLAC National Accelerator Laboratory, Menlo Park, CA 94025, USA
[6]Linac Coherent Lightsource, SLAC National Accelerator Laboratory, Menlo Park, CA, 94025, USA
[7]Department of Chemistry, Fujita Health University and School of Health Sciences, Toyoake, Aichi, Japan
*Corresponding author: nicholas.edwards@manchester.ac.uk

Keywords: Synchrotron, X-ray, fluorescence, absorption, melanin, feather, fossil

Feathers

All feathers were ethically sourced from captive animals (unreleasable or rehabilitated wildlife). All feathers analysed at SSRL were obtained from animals under the care of the Live Animal Center (LAC) at The Academy of Natural Sciences of Drexel University (Philadelphia, PA, USA). The feathers were naturally dropped via molting and collected by staff of the LAC from the enclosures. Each feather was placed in a sealed bag and stored in a designated freezer until the date of shipment for analysis. VET# is a unique LAC number used to track all husbandry and care of the animal. All birds are fed with feeder chicks, rats and mice, supplemented with Vionate Vitamin/Mineral Supplement.

Feathers used in melanin quantification were again natural molts obtained from captive birds. Harris Hawk, Eurasian Kestrel and Barn Owl were sourced from the non-profit Wild Wings Birds of Prey education and rehabilitation centre which was located in Bents Home & Garden, Warrington, UK at the time of feather sampling, but has since relocated to White Moss Nursery and Garden Centre, Warrington, UK. The Red-tailed Hawk feather was collected from The Falconry Centre in Hagley, West Midlands, UK. All birds are fed with feeder chicks.

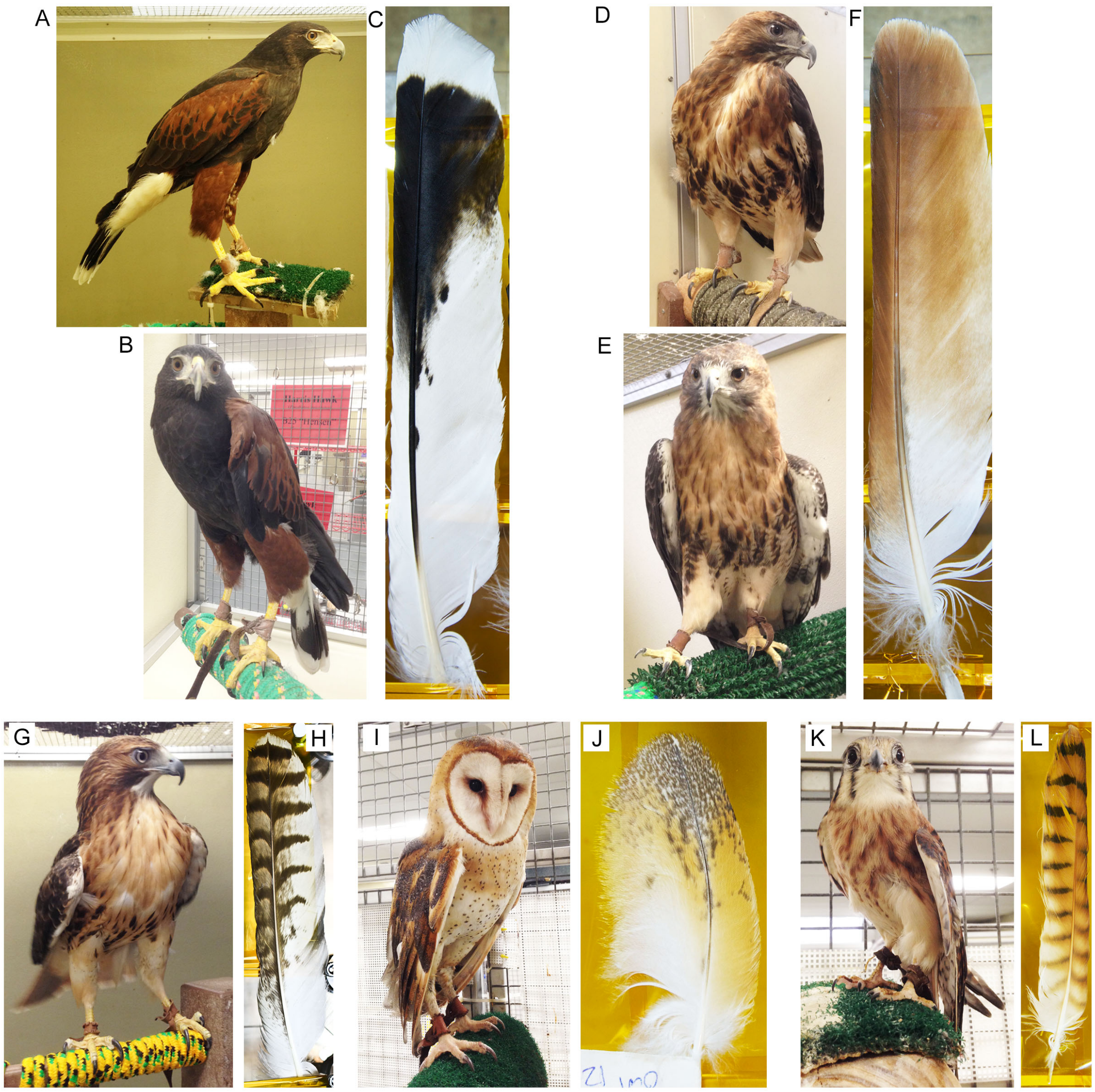

Species	Common Name	VET #	Call name	Sex	DOB/DOH	Age at acquisition	Date of Acquisition	Source
Parabuteo unicinctus	Harris Hawk	B25	Hensen	F	Unknown	Adult	2/19/1997	Adobe Mountain Wildlife Centre, AZ, USA
Parabuteo unicinctus	Harris Hawk	B69	Chase	F	Unknown	Adult	11/11/2009	Adobe Mountain Wildlife Centre, AZ, USA
Buteo jamaicensis	Red-tailed Hawk	B33	Teo	M	Unknown	Young adult	8/25/1997	Avian Rehabilitation, Weymouth, NJ, USA
Buteo jamaicensis	Red-tailed Hawk	B32	Mia	F	Unknown	Adult	8/25/1997	Avian Rehabilitation, Weymouth, NJ, USA
Buteo jamaicensis	Red-tailed Hawk	B72	Hawkeye	M	Spring 2012	Est. 1 year	3/17/2013	Centre Wildlife Care, Port Matilda, PA, USA
Tyto alba	Barn Owl	B66	Arizona	F	Unknown	Adult	11/11/2009	Adobe Mountain Wildlife Centre, AZ, USA
Falco sparverius	American Kestrel	B65	Sienna	F	Unknown	Adult	11/11/2009	Adobe Mountain Wildlife Centre, AZ, USA

Supplementary Figure 1. Optical images of the source animals and feathers from the LAC analysed at SSRL. (A) Harris Hawk, VET# B25, (B) Harris Hawk, VET# B69, (C) analysed tail feather, sourced from either B25 or B69, uncertainty due to these individuals sharing an enclosure, collected January 2014. (D) Red-tailed Hawk VET# B32, (E) Red-tailed Hawk VET# B33, (F) analysed tail feather, sourced from either B32 or B33, uncertainty due to these individuals sharing an enclosure, collected August 2014. (G) subadult Red-tailed Hawk VET# B72, (H) analysed tail feather, collected January 2014. (I) Barn Owl, VET# B66, (J) analysed body feather. (K) American Kestrel VET# B65, (L) analysed tail feather, collected January 2014.

Supplementary Table 1. Melanin content of feathers. These analyses were performed according to the methods outlined in (1-4). Note that UK feathers were analysed with the red-tailed hawk being the purely red feather comparable to figure S1b. Correlation between TM (x) and EM+PM (y) is y= 0.961x+1.51 (R^2 = 0.966).

		Soluene-350 solubilization (ng/mg)		H2O2 oxidation (ng/mg)		HI hydrolysis (ng/mg)	Various ratios		
Sample	Color	A500	A650	PTCA	TTCA	4-AHP	A650/A500	TTCA/PTCA	4-AHP/PTCA
Harris Hawk	Dark	0.6295	0.2195	711	299	6.4	0.349	0.42	0.01
	White	0.0015	0.0005	0.0	11.8	2.9	Not applicable	Not available	Not available
Kestrel	Dark	0.482	0.1535	591	268	109	0.318	0.45	0.18
	Red	0.326	0.0425	117	1193	1170	0.130	10.20	10.00
Barn Owl	Dark	0.135	0.043	171	35.2	17.5	0.319	0.21	0.10
	Red	0.055	0.012	57.0	105	416	0.218	1.84	7.30
	White	0.0065	0.002	7.2	47.8	7.5	Not applicable	6.64	1.04
Red Tailed Hawk	Red	0.246	0.024	80.3	1161	1067	0.098	14.46	13.29
	White	0.006	0.0015	6.0	14.7	9.5	Not applicable	2.45	1.58

TTCA = thiazole-2,4,5-tricarboxylic acid

PTCA = pyrrole-2,3,5-tricarboxylic acid

4-AHP = 4-amino-3-hydroxyphenylalanine

Sample	Color	TM = A500x101* or 215** (μg/mg)	EM = PTCA x 0.08 (μg/mg)	BZ-PM = TTCA x 0.034 (μg/mg)	BH-PM = 4-AHP x 0.007 (μg/mg)	Melanin = EM + BZ-PH + BH-PM (μg/mg)	PM = (BZ-PM) + (BH-PM) (μg/mg)
Harris Hawk	Dark	63.58	56.88	10.17	0.04	67.09	10.21
	White	0.15	0.00	0.40	0.02	0.42	0.42
Kestrel	Dark	48.68	47.28	9.11	0.76	57.16	9.88
	Red	70.09	9.36	40.56	8.19	58.11	48.75
Barn Owl	Dark	13.64	13.68	1.20	0.12	15.00	1.32
	Red	11.83	4.56	3.57	2.91	11.04	6.48
	White	0.66	0.58	1.63	0.05	2.25	1.68
Red Tailed Hawk	Red	52.89	6.42	39.47	7.47	53.37	46.94
	White	0.61	0.48	0.50	0.07	1.05	0.57

* = for EM

** = for PM

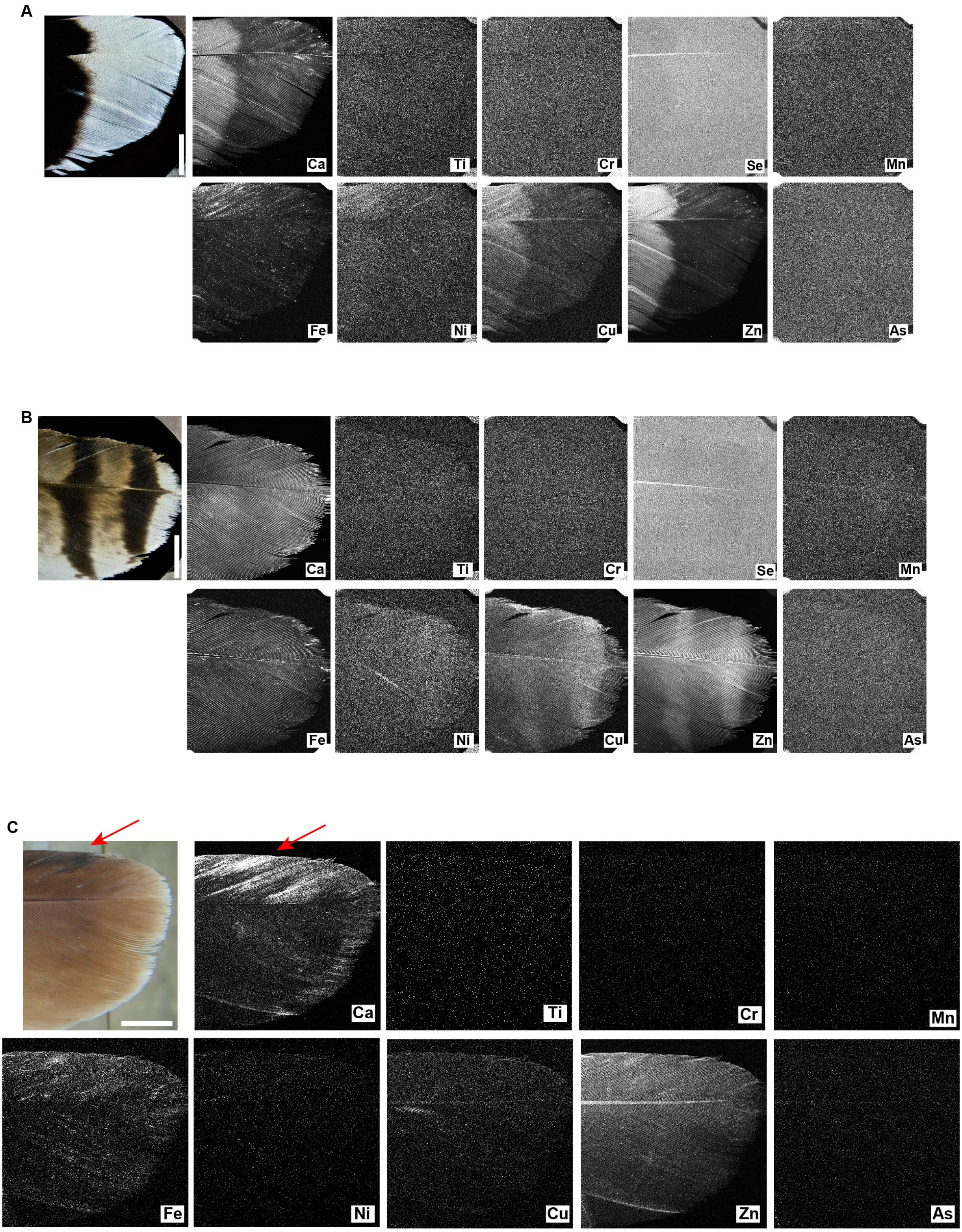

Supplementary Figure 2-1. SRS-XRF maps of all elements in A) Harris hawk, B) striped red-tailed hawk and C) non-striped red-tailed hawk. Red arrows indicate darker regions with high Ca. Scale bars = 1cm.

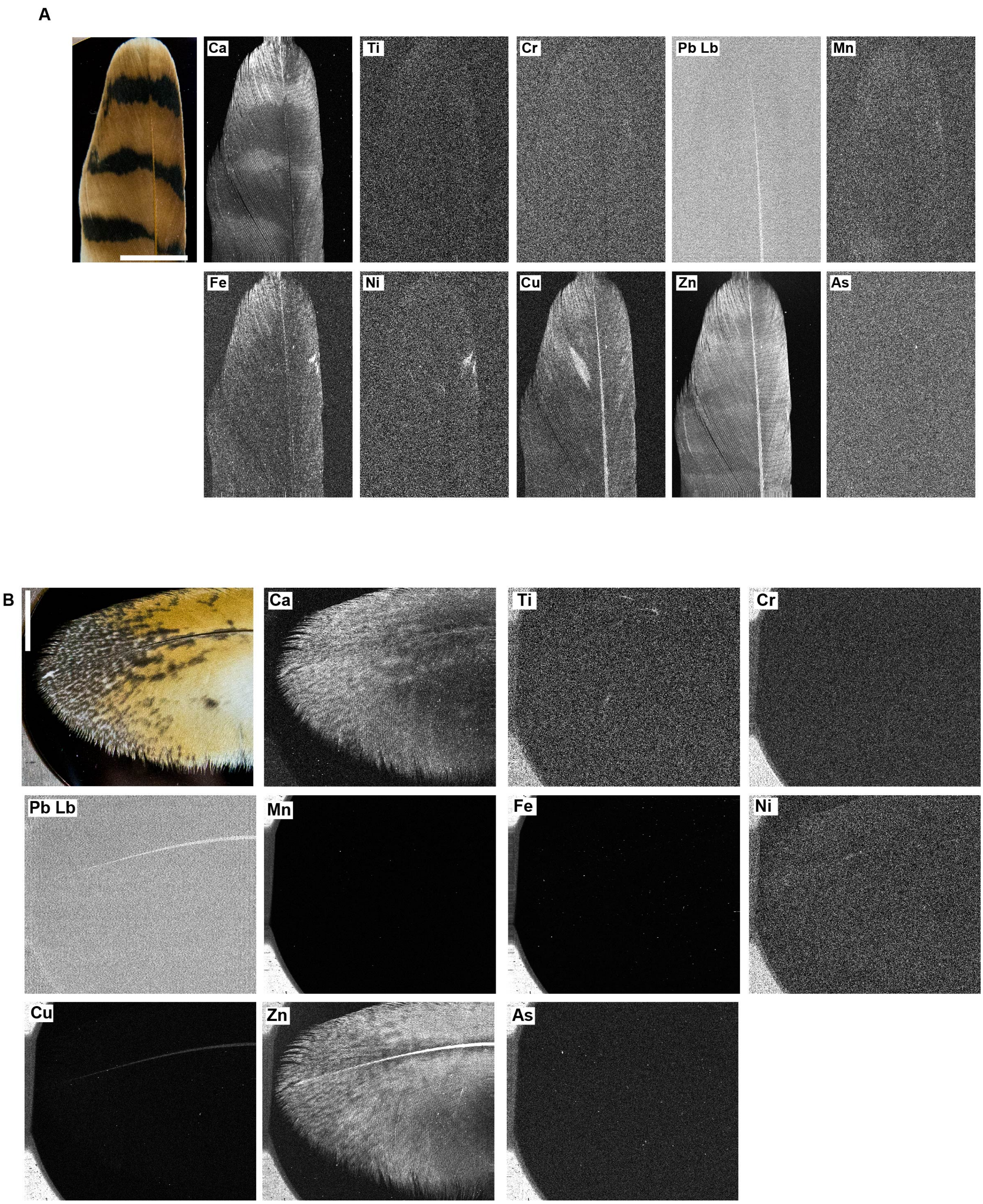

Supplementary Figure 2-2. SRS-XRF maps of all elements mapped in A) American kestrel and B) barn owl. Scale bars = 1 cm.

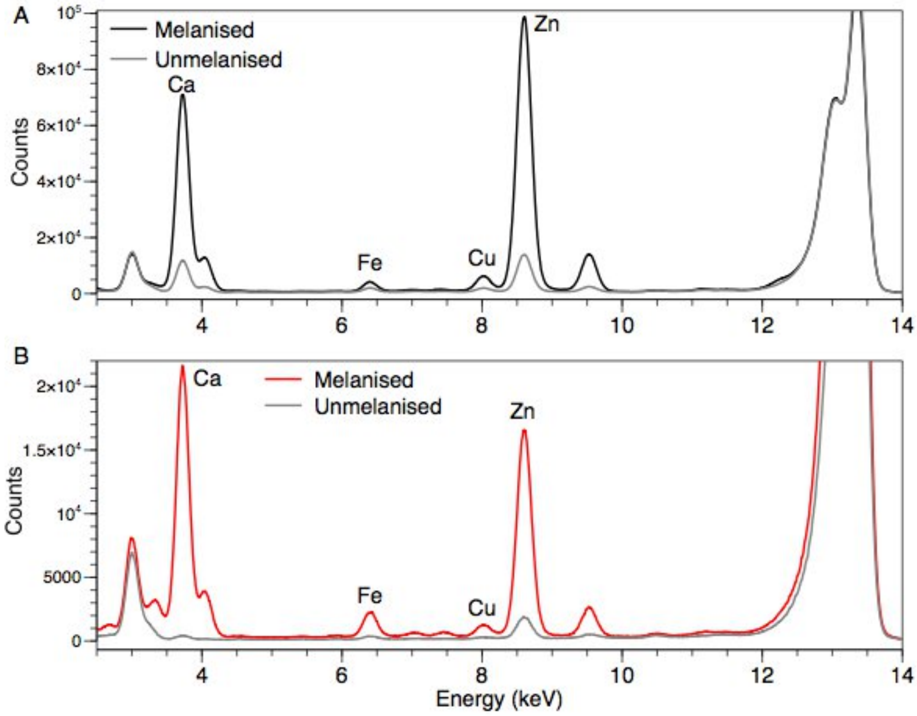

Supplementary Figure 3. Example synchrotron XRF EDS spectra of A) Harris Hawk and B) Red-tailed Hawk.

Supplementary Table 2. SXRF EDS of all elements detected within feathers. All values in parts per million (ppm) with 2σ errors in parantheses. Data fit using PyMCA (5) from fundamental parameters.

Species	Colour	S		Ca		Ti		Cr		Mn		Fe		Ni		Cu		Zn		As		Se	
**Harris hawk**	Black	33477	(1606)	6421	(389)	7	(1)	1	(1)	1	(1)	26	(2)	0		12	(1)	186	(10)	1	(1)	1	(1)
	White	25542	(1315)	1960	(159)	5	(1)	1	(1)	0		10	(1)	1	(1)	5	(1)	37	(3)	1	(1)	1	(1)
**kestrel**	Black	19423	(896)	1574	(123)	2	(1)	1	(1)	1	(1)	5	(1)	1	(1)	2	(1)	25	(2)	1	(1)	1	(1)
	Red	13550	(685)	989	(85)	2	(1)	1	(1)	1	(1)	5	(1)	1	(1)	3	(1)	49	(3)	1	(1)	1	(1)
**barn owl**	Black	11812	(515)	733	(73)	3	(1)	1	(1)	1	(1)	3	(1)	1	(1)	1	(1)	43	(3)	1	(1)	1	(1)
	Red	27833	(1005)	423	(47)	3	(1)	1	(1)	1	(1)	4	(1)	1	(1)	1	(1)	47	(3)	1	(1)	1	(1)
	White	12059	(521)	137	(18)	3	(1)	1	(1)	1	(1)	3	(1)	1	(1)	1	(1)	25	(2)	1	(1)	1	(1)
**red-tailed hawk**	Red	28853	(1181)	2060	(160)	2	(1)	1	(1)	1	(1)	8	(1)	1	(1)	2	(1)	32	(3)	0	(1)	1	(1)
	White	23343	(954)	35	(7)	2	(1)	1	(1)	0	(1)	1	(1)	0	(1)	0	(1)	6	(1)	0	(1)	0	(1)
**Striped red**	Brown	77307	(3793)	4315	(287)	6	(1)	1	(1)	2	(1)	31	(3)	1	(1)	17	(2)	226	(11)	1	(1)	1	(1)
**tailed hawk**	White	51813	(2801)	1144	(107)	3	(1)	0	(1)	1	(1)	6	(1)	0	(1)	4	(1)	31	(3)	1	(1)	1	(1)

Supplementary Figure 4. Representative fitting statistics exported by PyMCA.

Spectrum, Continuum and Fitted values :

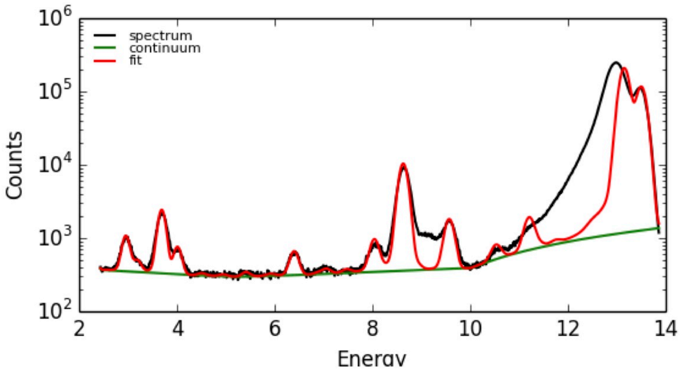

Fit Parameters :

FIT parameters	
Region of Fit	250 - 1380
Number of iterations	1
Chi square	6482.0627
Last Chi square difference	724046406.4801 %

Calibration parameters	
Zero	-8.79617E-02 +/- -8.79617E-02
Gain	1.01060E-02 +/- 1.01060E-02
Noise	1.50000E-01 +/- 1.50000E-01
Fano	1.20000E-01 +/- 1.20000E-01
Sum	0.00000E+00 +/- 0.00000E+00

Peak shape parameters	
ST AreaR	3.50000E-02 +/- 3.50000E-02
ST SlopeR	2.50000E-01 +/- 2.50000E-01
LT AreaR	0.00000E+00 +/- 0.00000E+00
LT SlopeR	1.00000E+01 +/- 1.00000E+01
STEP HeightR	0.00000E+00 +/- 0.00000E+00

Continuum parameters	
Type	Strip Background
Strip Constant	1.00000
Strip Iterations	20000
Strip Width	4
Smoothing Filter Width	10

Concentrations:

Element	Group	Fit Area	Sigma Area	M	a s s f r a c t i o n Layer0
Ar	K	1.637619e+04	1.72e+02	0.01602	0.01602
Ca	K	4.907065e+04	2.50e+02	0.0042	0.0042
Ti	K	5.415252e+02	1.11e+02	9.408e-06	9.408e-06
V	K	3.544807e+02	1.11e+02	3.314e-06	3.314e-06
Cr	K	1.039197e+03	1.14e+02	5.544e-06	5.544e-06
Mn	K	3.512201e+02	1.12e+02	1.205e-06	1.205e-06
Fe	K	8.645846e+03	1.48e+02	1.964e-05	1.964e-05
Ni	K	9.899093e+02	1.18e+02	1.268e-06	1.268e-06
Cu	K	1.523698e+04	1.73e+02	1.582e-05	1.582e-05
Zn	K	2.638458e+05	5.22e+02	0.0002191	0.0002191
As	K	8.293948e+03	1.69e+02	4.543e-06	4.543e-06
Se	K	3.494618e+04	2.47e+02	1.791e-05	1.791e-05

Fit Peak Results:

Element	Group	Fit Area	Sigma	Energy	Ratio	FWHM	Chi square
**Ar**	**K**	**1.637619e+04**	**1.72e+02**				
	KL3	1.354776e+04	1.47e+02	2.957	0.82728	0.173	5.17
	KM3	2.828429e+03	3.07e+01	3.190	0.17272	0.175	3.43
	KL3 Si_KM3esc	4.544048e+00	4.93e-02	1.121	0.00035	0.159	0.00
	KL3 Si_KL3esc	1.547234e+02	1.68e+00	1.217	0.01182	0.160	0.00
	KM3 Si_KM3esc	8.761060e-01	9.51e-03	1.354	0.00032	0.161	0.00
	KM3 Si_KL3esc	2.965263e+01	3.22e-01	1.450	0.01085	0.162	0.00
**Ca**	**K**	**4.907065e+04**	**2.50e+02**				
	KL3	4.045270e+04	2.13e+02	3.690	0.82438	0.179	35.27
	KM3	8.617950e+03	4.54e+01	4.013	0.17562	0.181	22.12
	KL3 Si_KM3esc	1.048440e+01	5.52e-02	1.855	0.00027	0.165	0.00
	KL3 Si_KL3esc	3.505733e+02	1.85e+00	1.951	0.00897	0.166	0.00
	KM3 Si_KM3esc	1.985784e+00	1.05e-02	2.177	0.00024	0.168	0.00
	KM3 Si_KL3esc	6.593018e+01	3.47e-01	2.273	0.00792	0.168	0.53
**Ti**	**K**	**5.415252e+02**	**1.11e+02**				
	KL3	4.545733e+02	9.67e+01	4.509	0.83943	0.185	0.97
	KM3	8.695189e+01	1.85e+01	4.932	0.16057	0.187	1.02
	KL3 Si_KM3esc	8.682059e-02	1.85e-02	2.673	0.00020	0.171	2.04
	KL3 Si_KL3esc	2.853917e+00	6.07e-01	2.769	0.00650	0.172	3.08
	KM3 Si_KM3esc	1.416038e-02	3.01e-03	3.096	0.00017	0.174	4.04
	KM3 Si_KL3esc	4.620851e-01	9.83e-02	3.192	0.00550	0.175	3.43
**V**	**K**	**3.544807e+02**	**1.11e+02**				
	KL3	2.997193e+02	9.75e+01	4.950	0.84552	0.188	1.04
	KM3	5.476141e+01	1.78e+01	5.427	0.15448	0.191	1.41
	KL3 Si_KM3esc	4.848483e-02	1.58e-02	3.114	0.00017	0.175	4.05
	KL3 Si_KL3esc	1.581719e+00	5.15e-01	3.210	0.00546	0.175	3.65
	KM3 Si_KM3esc	7.394894e-03	2.41e-03	3.591	0.00014	0.178	21.60
	KM3 Si_KL3esc	2.395398e-01	7.79e-02	3.688	0.00453	0.179	35.27
**Cr**	**K**	**1.039197e+03**	**1.14e+02**				
	KL3	8.891340e+02	1.01e+02	5.412	0.85560	0.191	1.42
	KM3	1.500625e+02	1.70e+01	5.947	0.14440	0.194	1.08
	KL3 Si_KM3esc	1.207762e-01	1.37e-02	3.576	0.00014	0.178	19.03
	KL3 Si_KL3esc	3.913088e+00	4.43e-01	3.672	0.00456	0.179	35.04
	KM3 Si_KM3esc	1.672441e-02	1.89e-03	4.111	0.00012	0.182	5.86
	KM3 Si_KL3esc	5.383056e-01	6.10e-02	4.207	0.00371	0.182	2.66
**Mn**	**K**	**3.512201e+02**	**1.12e+02**				
	KL2	1.012070e+02	3.34e+01	5.888	0.28816	0.194	1.15
	KL3	1.995340e+02	6.58e+01	5.899	0.56812	0.194	1.14
	KM3	5.047908e+01	1.67e+01	6.490	0.14372	0.198	2.04
	KL2 Si_KM3esc	1.152514e-02	3.80e-03	4.052	0.00012	0.181	17.70
	KL2 Si_KL3esc	3.712030e-01	1.22e-01	4.148	0.00380	0.182	2.72
	KL3 Si_KM3esc	2.263043e-02	7.47e-03	4.063	0.00012	0.181	15.10
	KL3 Si_KL3esc	7.287918e-01	2.40e-01	4.159	0.00378	0.182	2.61
	KM3 Si_KM3esc	4.616405e-03	1.52e-03	4.654	0.00009	0.186	1.00
	KM3 Si_KL3esc	1.477731e-01	4.87e-02	4.751	0.00303	0.186	0.93
**Fe**	**K**	**8.645846e+03**	**1.48e+02**				
	KL2	2.508388e+03	4.43e+01	6.391	0.29013	0.197	1.74
	KL3	4.933868e+03	8.72e+01	6.404	0.57066	0.197	1.77
	KM3	1.203590e+03	2.13e+01	7.058	0.13921	0.201	1.57
	KL2 Si_KM3esc	2.377360e-01	4.20e-03	4.555	0.00010	0.185	1.02
	KL2 Si_KL3esc	7.617031e+00	1.35e-01	4.651	0.00314	0.186	1.00
	KL3 Si_KM3esc	4.654310e-01	8.22e-03	4.568	0.00010	0.185	1.00
	KL3 Si_KL3esc	1.491053e+01	2.63e-01	4.664	0.00313	0.186	0.97
	KM3 Si_KM3esc	9.023697e-02	1.59e-03	5.222	0.00008	0.189	1.26
	KM3 Si_KL3esc	2.875295e+00	5.08e-02	5.318	0.00247	0.190	1.48
**Ni**	**K**	**9.899093e+02**	**1.18e+02**				
	KL2	2.895296e+02	3.57e+01	7.461	0.29248	0.204	1.35
	KL3	5.676757e+02	6.99e+01	7.478	0.57346	0.204	1.32
	KM3	1.327040e+02	1.64e+01	8.265	0.13406	0.209	53.45
	KL2 Si_KM3esc	1.895168e-02	2.34e-03	5.625	0.00007	0.192	1.28
	KL2 Si_KL3esc	6.022586e-01	7.42e-02	5.721	0.00215	0.193	1.32
	KL3 Si_KM3esc	3.694716e-02	4.55e-03	5.642	0.00007	0.192	1.32
	KL3 Si_KL3esc	1.174007e+00	1.45e-01	5.738	0.00214	0.193	1.35
	KM3 Si_KM3esc	6.701900e-03	8.26e-04	6.429	0.00005	0.197	1.83
	KM3 Si_KL3esc	2.120745e-01	2.61e-02	6.525	0.00165	0.198	1.97
**Cu**	**K**	**1.523698e+04**	**1.73e+02**				
	KL2	4.492044e+03	5.27e+01	8.028	0.29481	0.208	14.27
	KL3	8.779109e+03	1.03e+02	8.048	0.57617	0.208	15.98
	KM3	1.965827e+03	2.30e+01	8.905	0.12902	0.213	415.52

	KL2 Si_KM3esc	2.446812e-01	2.87e-03	6.192	0.00006	0.196	1.32
	KL2 Si_KL3esc	7.751449e+00	9.09e-02	6.288	0.00179	0.196	1.48
	KL3 Si_KM3esc	4.751405e-01	5.57e-03	6.212	0.00006	0.196	1.36
	KL3 Si_KL3esc	1.505086e+01	1.76e-01	6.308	0.00177	0.197	1.61
	KM3 Si_KM3esc	8.150738e-02	9.55e-04	7.069	0.00004	0.202	1.58
	KM3 Si_KL3esc	2.572532e+00	3.02e-02	7.166	0.00135	0.202	1.44
**Zn**	**K**	**2.638458e+05**	**5.22e+02**				
	KL2	7.799260e+04	1.60e+02	8.616	0.29560	0.211	223.16
	KL3	1.521468e+05	3.12e+02	8.639	0.57665	0.211	246.72
	KM3	3.370635e+04	6.91e+01	9.572	0.12775	0.217	78.91
	KL2 Si_KM3esc	3.530617e+00	7.24e-03	6.780	0.00005	0.200	1.86
	KL2 Si_KL3esc	1.115552e+02	2.29e-01	6.876	0.00148	0.200	1.68
	KL3 Si_KM3esc	6.838841e+00	1.40e-02	6.803	0.00005	0.200	1.83
	KL3 Si_KL3esc	2.160637e+02	4.43e-01	6.899	0.00147	0.200	1.71
	KM3 Si_KM3esc	1.150616e+00	2.36e-03	7.736	0.00004	0.206	3.94
	KM3 Si_KL3esc	3.623893e+01	7.43e-02	7.832	0.00111	0.206	8.33
**As**	**K**	**8.293948e+03**	**1.69e+02**				
	KL2	2.462016e+03	5.19e+01	10.508	0.29684	0.222	10.21
	KL3	4.776126e+03	1.01e+02	10.544	0.57586	0.223	12.03
	KM3	1.018426e+03	2.15e+01	11.724	0.12279	0.229	1914.30
	KN3	3.737964e+01	7.88e-01	11.864	0.00451	0.230	3243.29
	KL2 Si_KM3esc	6.490481e-02	1.37e-03	8.672	0.00003	0.211	267.49
	KL2 Si_KL3esc	2.039575e+00	4.30e-02	8.768	0.00086	0.212	324.71
	KL3 Si_KM3esc	1.247184e-01	2.63e-03	8.708	0.00003	0.212	283.36
	KL3 Si_KL3esc	3.918879e+00	8.26e-02	8.804	0.00085	0.212	356.94
	KM3 Si_KM3esc	1.966401e-02	4.14e-04	9.888	0.00002	0.219	12.34
	KM3 Si_KL3esc	6.166478e-01	1.30e-02	9.984	0.00063	0.219	10.85
	KN3 Si_KM3esc	6.975801e-04	1.47e-05	10.028	0.00002	0.220	7.06
	KN3 Si_KL3esc	2.187140e-02	4.61e-04	10.124	0.00061	0.220	3.09
**Se**	**K**	**3.494618e+04**	**2.47e+02**				
	KL2	1.038191e+04	7.58e+01	11.182	0.29708	0.226	119.41
	KL3	2.008707e+04	1.47e+02	11.222	0.57480	0.226	159.66
	KM3	4.246857e+03	3.10e+01	12.494	0.12153	0.233	43200.32
	KN3	2.303504e+02	1.68e+00	12.652	0.00659	0.234	89928.33
	KL2 Si_KM3esc	2.295130e-01	1.68e-03	9.346	0.00002	0.216	250.53
	KL2 Si_KL3esc	7.203262e+00	5.26e-02	9.442	0.00072	0.216	169.85
	KL3 Si_KM3esc	4.395272e-01	3.21e-03	9.386	0.00002	0.216	215.24
	KL3 Si_KL3esc	1.379364e+01	1.01e-01	9.482	0.00071	0.216	139.86
	KM3 Si_KM3esc	6.829516e-02	4.99e-04	10.658	0.00002	0.223	20.90
	KM3 Si_KL3esc	2.139648e+00	1.56e-02	10.754	0.00052	0.224	24.98
	KN3 Si_KM3esc	3.571925e-03	2.61e-05	10.816	0.00002	0.224	23.37
	KN3 Si_KL3esc	1.118874e-01	8.17e-04	10.912	0.00050	0.225	41.58
**Scatter**	**Peak000**	**2.968575e+06**	**1.77e+03**				
	Scatter 000	2.968575e+06	1.83e+03	13.500	1.00000	0.239	3103.33
	Scatter 000 Si_KM3esc	3.814037e+01	2.35e-02	11.664	0.00001	0.229	1530.88
	Scatter 000 Si_KL3esc	1.193775e+03	7.36e-01	11.760	0.00042	0.229	2239.53
**Scatter**	**Compton000**	**5.268037e+06**	**2.29e+03**				
	Scatter 000	5.268037e+06	2.37e+03	13.153	1.00000	0.237	58313.61
	Scatter 000 Si_KM3esc	7.300999e+01	3.29e-02	11.317	0.00001	0.227	297.12
	Scatter 000 Si_KL3esc	2.285861e+03	1.03e+00	11.413	0.00045	0.227	498.52

Spectrum, Continuum and Fitted values :

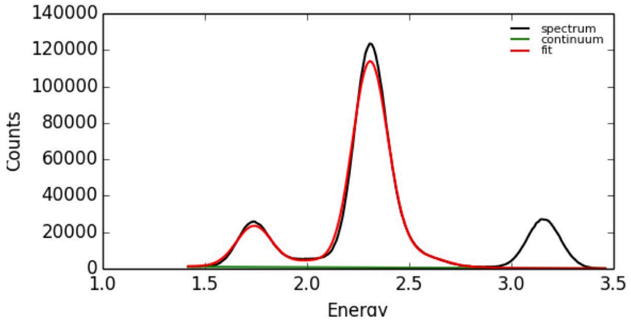

Fit Parameters :

FIT parameters	
Region of Fit	150 - 350
Number of iterations	2
Chi square	2847.9592
Last Chi square difference	56104797.1259 %

Calibration parameters	
Zero	-1.12093E-01 +/- -1.12093E-01
Gain	1.02033E-02 +/- 1.02033E-02
Noise	2.00000E-01 +/- 2.00000E-01
Fano	1.20000E-02 +/- 1.20000E-02
Sum	0.00000E+00 +/- 0.00000E+00

Peak shape parameters	
ST AreaR	3.50000E-02 +/- 3.50000E-02
ST SlopeR	2.50000E-01 +/- 2.50000E-01
LT AreaR	0.00000E+00 +/- 0.00000E+00
LT SlopeR	1.00000E+01 +/- 1.00000E+01
STEP HeightR	0.00000E+00 +/- 0.00000E+00

Continuum parameters	
Type	Strip Background
Strip Constant	1.00000
Strip Iterations	20000
Strip Width	4
Smoothing Filter Width	10

Concentrations:

Element	Group	Fit Area	Sigma Area	M	a s s f r a c t i o n	Layer0
Al	K	0.000000e+00	0.00e+00	0	0	
Si	K	1.385655e+04	2.35e+02	0.0008861	0.0008861	
P	K	2.594310e+04	3.28e+02	0.0006497	0.0006497	
S	K	1.947235e+06	1.40e+03	0.02129	0.02129	
Cl	K	8.586620e+04	3.81e+02	0.0006206	0.0006206	

Fit Peak Results:

Element	Group	Fit Area	Sigma	Energy	Ratio	FWHM	Chi square
**Al**	**K**	**0.000000e+00**	**0.00e+00**				
	KL3	0.000000e+00	0.00e+00	1.487	0.98330	0.201	328.42
	KM3	0.000000e+00	0.00e+00	1.554	0.01670	0.201	285.91
**Si**	**K**	**4.845518e+05**	**7.30e+02**				
	KL3	4.682508e+05	7.30e+02	1.740	0.96636	0.201	210.75
	KM3	1.630108e+04	2.54e+01	1.836	0.03364	0.201	106.85
**P**	**K**	**5.056266e+04**	**4.30e+02**				
	KL3	4.795549e+04	4.22e+02	2.010	0.94844	0.201	363.34
	KM3	2.607174e+03	2.29e+01	2.136	0.05156	0.201	473.99
	KL3 Si_KM3esc	2.132240e+01	1.88e-01	0.174	0.00046	0.200	0.00
	KL3 Si_KL3esc	7.440874e+02	6.55e+00	0.270	0.01606	0.200	0.00
	KM3 Si_KM3esc	1.120838e+00	9.86e-03	0.300	0.00044	0.200	0.00
	KM3 Si_KL3esc	3.898702e+01	3.43e-01	0.396	0.01548	0.200	0.00
**S**	**K**	**2.574437e+06**	**1.60e+03**				
	KL3	2.399178e+06	1.55e+03	2.307	0.93192	0.201	449.11
	KM3	1.752593e+05	1.13e+02	2.464	0.06808	0.202	136.63
	KL3 Si_KM3esc	9.828586e+02	6.33e-01	0.471	0.00042	0.200	0.00
	KL3 Si_KL3esc	3.403453e+04	2.19e+01	0.568	0.01468	0.200	0.00
	KM3 Si_KM3esc	6.857617e+01	4.42e-02	0.628	0.00040	0.200	0.00
	KM3 Si_KL3esc	2.364930e+03	1.52e+00	0.724	0.01397	0.200	0.00
**Cl**	**K**	**9.385624e+04**	**4.02e+02**				
	KL3	8.579319e+04	3.80e+02	2.622	0.91409	0.202	19.04
	KM3	8.063052e+03	3.57e+01	2.816	0.08591	0.202	1603.67
	KL3 Si_KM3esc	3.199878e+01	1.42e-01	0.786	0.00039	0.201	0.00
	KL3 Si_KL3esc	1.098969e+03	4.87e+00	0.882	0.01326	0.201	0.00
	KM3 Si_KM3esc	2.830048e+00	1.25e-02	0.980	0.00036	0.201	0.00
	KM3 Si_KL3esc	9.670925e+01	4.29e-01	1.076	0.01241	0.201	0.00

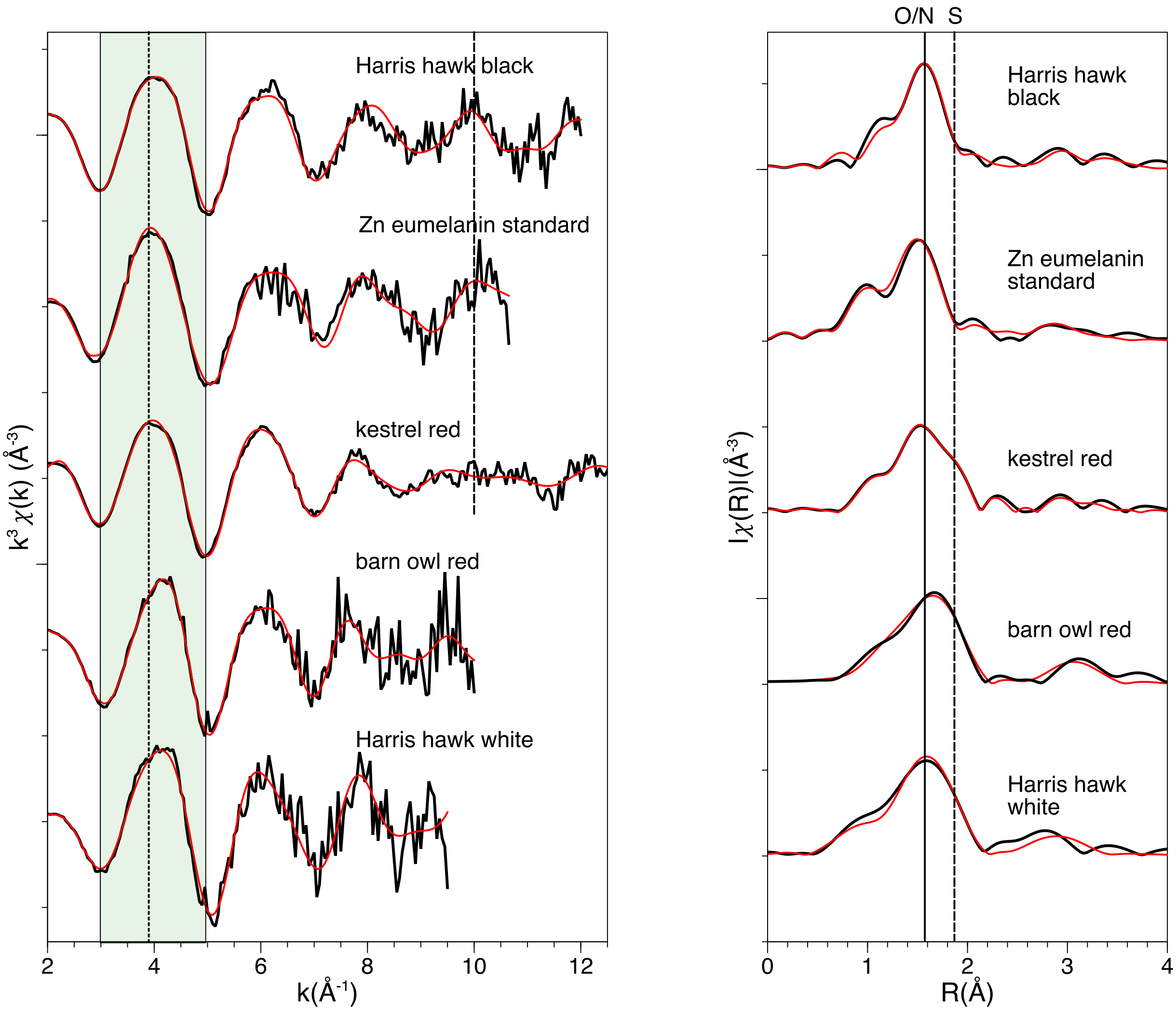

Supplementary Figure 5. Zn K-edge EXAFS spectra k^3-weighted (left) and their Fourier transform (right) of feathers. The Hamilton and F-tests were used to verify the significance of all shells in the fitted models. The k^3-weighted data ranges (left) represent the range of the fitting window of the fitted model. Green vertical bar indicates the shifted peak between feathers with a eumelanin component compared to feathers that are predominantly pheomelanised. Dotted line in the green bar represents the peak shift. Dashed line indicates the presence of a strong peak at $k=10Å^{-1}$ in both the Harris hawk black and Zn eumelanin standard, which is absent in the pheomelanised kestrel feather.
The dominant peak in the RDF (right) represents O/N (solid vertical line), while the presence of S is indicated by a distal shoulder on this peak (indicated by the vertical dashed line).
Due to the extremely low concentrations of Zn in the barn owl and white portion of the Harris hawk feathers, the fitting range is limited for those measurements. Once Fourier transformed however, these are also consistent with first shell sulfur.

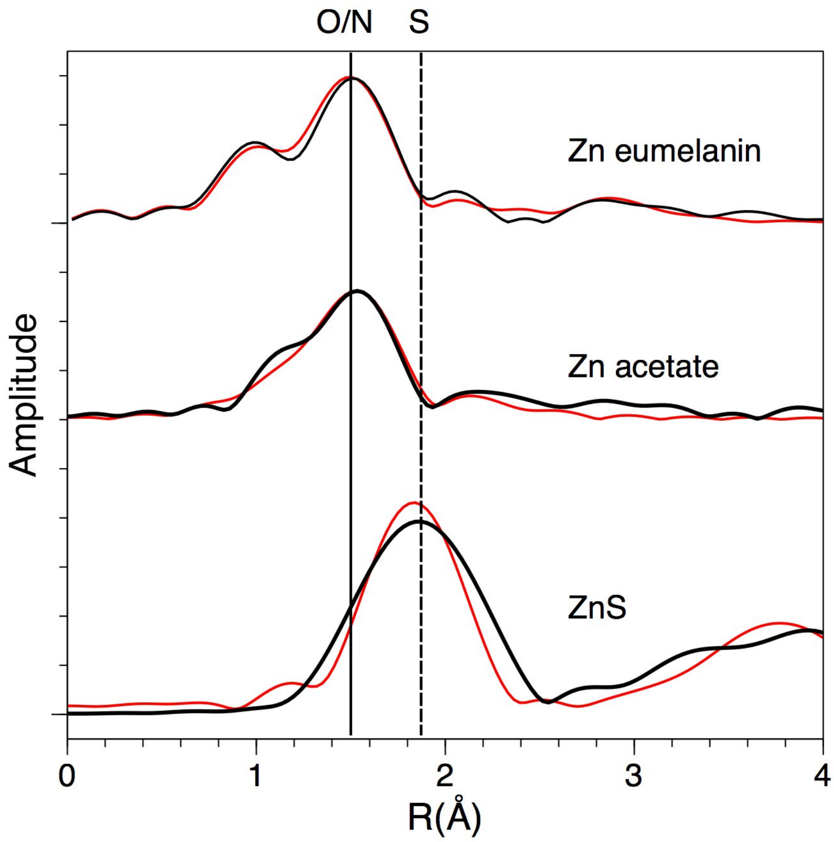

Supplementary Figure 6. Zn K-edge EXAFS Fourier transform of the Zn standards: Zn doped eumelanin, Zn acetate, and Zn sulfide (wurtzite). The dominant peak in the eumelanin and acetate represents O/N (solid vertical line). This shows that Zn-acetate is a well-constrained Zn-O coordination environment, and a ZnS standard provides constraints on Zn-S bonds. The dominant peak in the eumelanin and acetate represents O/N (solid vertical line), while the dominant S is indicated by a distal shoulder on this peak. This shows that the Zn-eumelanin is purely light element coordinated (O/N, solid vertical line) as in the Zn-acetate standard. The dominant peak in the ZnS standard (indicated by the vertical dashed line), is due to sulfur in the first shell, and is directly comparable to the distal shoulder on the dominant red kestrel peak in Figure S5 (also vertical dashed line) and is a clear indicator that Zn in the kestrel feather is bound to first shell sulfur. Because the chemical degradation studies show that the red kestrel feathers are over 80% pheomelanin, we interpret this as due to pheomelanin complexation.

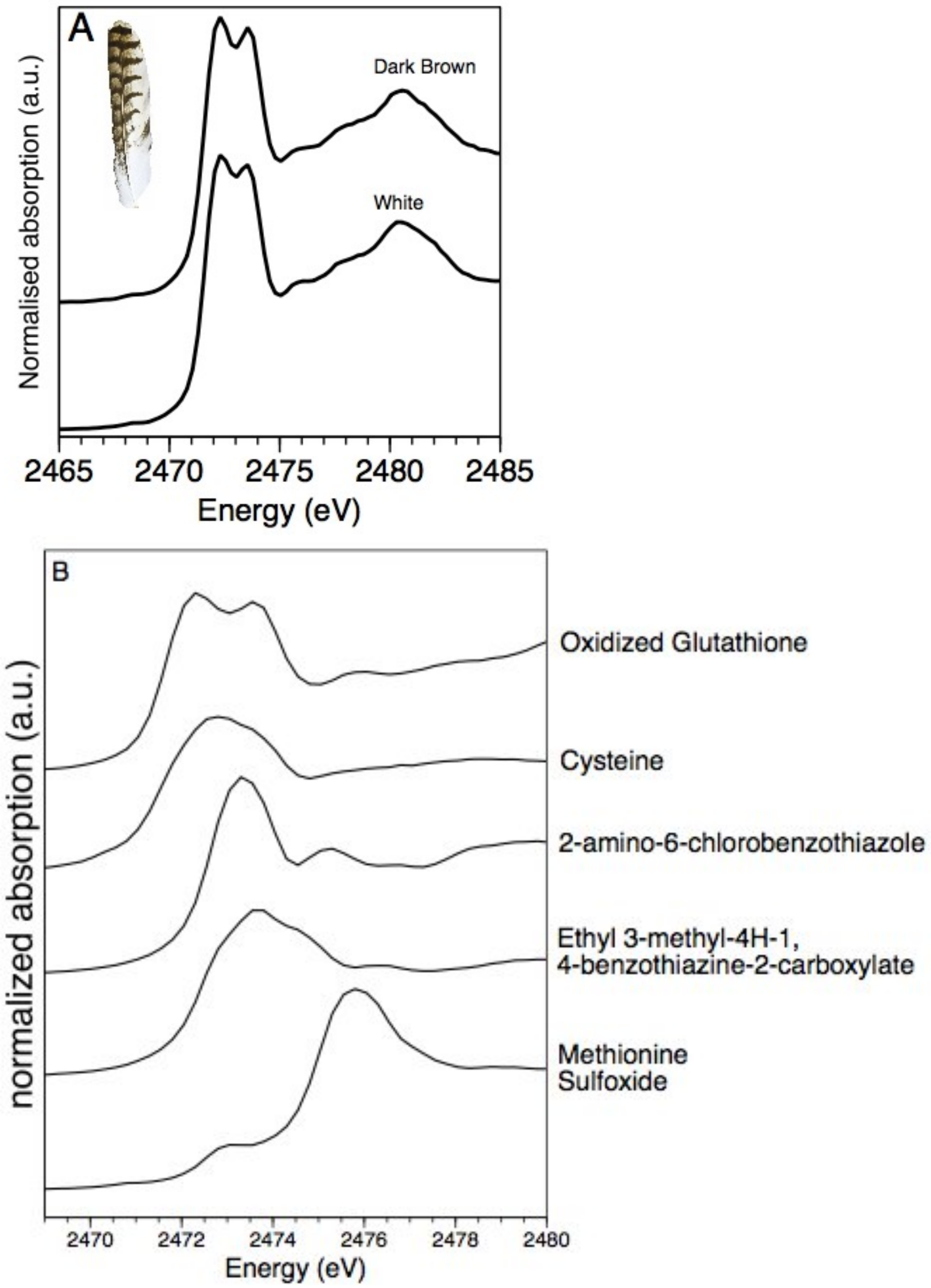

Supplementary Figure 7. S XANES data for A) striped red-tailed hawk, B) sulfur standards.

References

1. Ozeki H., Ito S., Wakamatsu K., Thody A.J. Spectrophotometric characterization of eumelanin and pheomelanin in hair. *Pigm. Cell Res.* **9**, 265-270 (1996).
2. Wakamatsu K., Ito S., Rees J.L. The usefulness of 4-amino-3-hydroxyphenylalanine as a specific marker of pheomelanin. *Pigm. Cell Res.* **15**:225-232 (2002).
3. Ito S*., et al.* Usefulness of alkaline hydrogen peroxide oxidation to analyze eumelanin and pheomelanin in various tissue samples: application to chemical analysis of human hair melanins. *Pigm Cell. Melanoma Res.* **24**, 605-613 (2011).
4. Hirobe T, Ito S, Wakamatsu K. The mouse ruby-eye 2d (ru2d/Hps5ru2-d) allele inhibits eumelanin but not pheomelanin synthesis. *Pigm Cell Melanoma Res* **26**, 723-726 (2013).
5. Solé V.A., Papillon E., Cotte M., Walter P., Susini J. A multiplatform code for the analysis of energy-dispersive X-ray fluorescence spectra. *Spectrochim. Acta B* **62**, 63-68 (2007).
